# Supplementary material for: Resistance pattern of infected chronic wound isolates and factors associated with bacterial resistance to third generation cephalosporins at Mbarara Regional Referral Hospital, Uganda
Source: PLoS One. 2021 Dec 16;16(12):e0261264. doi: 10.1371/journal.pone.0261264 (PMC8675733; doi:10.1371/journal.pone.0261264)
Supplement: Attachment — Submitted filename: Response _to_ Reviewers.docx [file pone.0261264.s002.docx]

**Response to Reviewers:**

**Preamble:** this document outlines the changes/revisions made as a result of review process made. All revisions have been written in red colour. This document consists of two main sections (journal requirements and Review Comments to the Author). The authors have also made the following minor changes;

-The phrase ‘’infected chronic wound’’ is changed to “chronic wound infection” throughout the revised manuscript.

- Additional affiliation (5) was added in the revised manuscript were the first author works as a head of department.

(a) **Journal Requirements:**
1. *Please ensure that your manuscript meets PLOS ONE's style requirements, including those for file naming. The PLOS ONE style templates can be found at* [*https://journals.plos.org/plosone/s/file?id=wjVg/PLOSOne_formatting_sample_main_body.pdf*](https://journals.plos.org/plosone/s/file?id=wjVg/PLOSOne_formatting_sample_main_body.pdf) *and* [*https://journals.plos.org/plosone/s/file?id=ba62/PLOSOne_formatting_sample_title_authors_affiliations.pdf*](https://journals.plos.org/plosone/s/file?id=ba62/PLOSOne_formatting_sample_title_authors_affiliations.pdf)*.*

**Response:** All major section headings are level headings; bold with font size 18 throughout the revised manuscript, sub-headings are level 2 headings, bold and font size 16 while headings under subheadings are level 3 headings, bold and font size 14. All first letters of the headings are now capitalized. We also introduced cells in table 4 as per the above guidelines. See page 16. Equations have been restated using equation tool (page 6-7). Figure 1 has been removed from the manuscript body and submitted separately. Figure title and legend have been stated on page 14.

Title, authors and affiliations have been revised as per PLOS ONE guidelines above.

*2. Thank you for stating the following financial disclosure:*

*“This study had no funding support from any organization/Institution. The corresponding author used out-of- pocket to fund this study as part of  his postgraduate studies at Mbarara University of Science and Technology.*

*All the remaining authors did not receive any grant to support this study but contributed non-financial support to this study.”*

*At this time, please address the following queries:*

*a) Please clarify the sources of funding (financial or material support) for your study. List the grants or organizations that supported your study, including funding received from your institution.*

*b) State what role the funders took in the study. If the funders had no role in your study, please state: “The funders had no role in study design, data collection and analysis, decision to publish, or preparation of the manuscript.”*

*c) If any authors received a salary from any of your funders, please state which authors and which funders.*

*d) If you did not receive any funding for this study, please state: “The authors received no specific funding for this work.”*

**Response:** The authors have revised the above statement and the new statement of financial disclosure is “The authors received no specific funding for this work.” **See page 20** under declaration section.

3. *Please include your amended statements within your cover letter; we will change the online submission form on your behalf.*

**Response:** Amendments have been stated in the cover letter and Witten in red color in the cover letter.

4. *Thank you for stating the following in your Competing Interests section:*

*The following authors had no financial and non-financial competing interest;*

*1. W.K*

*2. J.M*

*3. T.M*

*4. T.S.S*

*Please complete your Competing Interests on the online submission form to state any Competing Interests. If you have no competing interests, please state ""The authors have declared that no competing interests exist."", as detailed online in our guide for authors at* [*http://journals.plos.org/plosone/s/submit-now*](http://journals.plos.org/plosone/s/submit-now)

**Response:** we have revised the above statement and the new statement is “The authors have declared that no competing interests exist” see competing interest on **page 20** of the revised manuscript with track changes highlighted in red color.

5. *PLOS requires an ORCID iD for the corresponding author in Editorial Manager on papers submitted after December 6th, 2016. Please ensure that you have an ORCID iD and that it is validated in Editorial Manager. To do this, go to ‘Update my Information’ (in the upper left-hand corner of the main menu), and click on the Fetch/Validate link next to the ORCID field. This will take you to the ORCID site and allow you to create a new iD or authenticate a pre-existing iD in Editorial Manager. Please see the following video for instructions on linking an ORCID iD to your Editorial Manager account:* [*https://www.youtube.com/watch?v=_xcclfuvtxQ*](https://www.youtube.com/watch?v=_xcclfuvtxQ)

**Response: T**he corresponding author has authenticated his pre-existing ID in the editorial manager. **The ORCID ID is 0000-0002-5446-9352.**

6. *Your ethics statement should only appear in the Methods section of your manuscript. If your ethics statement is written in any section besides the Methods, please delete it from any other section.*

**Response: T**he ethics statement has been removed **‘’**declaration sections’’ and ‘’selection criteria’’ and now appears only in methods section of the revised manuscript with track changes on **page 4** under methods section.

7. *Please review your reference list to ensure that it is complete and correct. If you have cited papers that have been retracted, please include the rationale for doing so in the manuscript text, or remove these references and replace them with relevant current references. Any changes to the reference list should be mentioned in the rebuttal letter that accompanies your revised manuscript. If you need to cite a retracted article, indicate the article’s retracted status in the References list and also include a citation and full reference for the retraction notice.*

***Response:*** Reference list has been reviewed. It is complete and correct.

**(b) Review Comments to the Author**

1. *While the study objectives are listed in the abstract there is no mention of the objectives in the methodology sections. It is not clear whether the authors differentiated between hospital or community acquired chronic wound infections given that this may influence antibiotic resistance patterns.*

**Response:** We (the authors) have clarified **on page 5** under study population we studied community acquired chronic wound infection. This has been stated also in objective 1 on **page 12** under subsection “data processing and analysis plan”

2. *While there was a consideration for ≤2 prior hospitalizations it is not clear whether “0” or no hospitalization was also evaluated.*

**Response:** we only considered patients with at least 1 hospitalization in a year. See a caption under **table 4** has been included to clarify this on **page 16.**

3. *While the background section mentions some comorbidities as factors contributing to bacterial resistance the authors do not state what comorbidities the study participants were evaluated for.*

**Response:** We studied patients with chronic wound infection who had **diabetes mellitus** and **hypertension.** This has now been included under subsection “selection criteria’’ on **page 6** of the revised manuscript.

4. *There are a few editorial issues that could be easily resolved by the authors e.g some minor typos with regard to the use of upper or lower case letters with regard to nouns.*

**Response:** We have made various corrections regarding minor typos with regard to the use of upper or lower case letters with regard to nouns, particularly names of drugs, isolates and other nouns throughout the revised manuscript. See highlighted the first letters of various nouns that have been capitalized e.g. **page 1** under subsection ’’results’’ of the abstract and **page 14** under subheading “resistance pattern of chronic wound isolates”

5. *Another grammatical error that could be corrected is in paragraph 2 of the section titled “Factors associated with bacterial resistance to the third generation cephalosporins”. The first sentence of in this second parapraph reads “Higher odds of bacterial resistance to more 2 brands of the third generation cephalosporins….”. Did the authors mean to sate “2 brands or more”, or “more than 2 brands”? This should be clarified.*

**Response:** The grammatical error has been resolved. We have stated “more than 2 brands” see **paragraph 2 on page 15** under subsection “factors associated with bacterial resistance to third generation cephalosporins”
